# Improving hand hygiene practices in two regional hospitals in Kenya using a continuous quality improvement (CQI) approach

**DOI:** 10.1186/s13756-022-01093-z

**Published:** 2022-04-04

**Authors:** Jemima Kibira, Loyce Kihungi, Mary Ndinda, Evelyn Wesangula, Catherine Mwangi, Faith Muthoni, Orvalho Augusto, George Owiso, Linus Ndegwa, Ulzii-Orshikh Luvsansharav, Elizabeth Bancroft, Peter Rabinowitz, John Lynch, Anne Njoroge

**Affiliations:** 1 International Training and Education Center for Health, P.O. Box 2614-00202, Nairobi, Kenya; 2 Department of Patient and Healthcare Worker Safety, Ministry of Health, Nairobi, Kenya; 3 Infection Prevention and Control Department, Thika Level 5 Hospital, Kiambu, Kenya; 4 Infection Prevention and Control Department, Kitale County Referral Hospital, Trans-Nzoia, Kenya; 5 Department of Global Health, University of Washington, Seattle, USA; 6 Division of Global Health Protection (DGHP), Center for Global Health (CGH), US Centers for Disease Control and Prevention (CDC), Nairobi, Kenya; 7 Division of Healthcare Quality Promotion, National Center for Emerging and Zoonotic Infectious Diseases (NCEZID), CDC, Atlanta, GA USA; 8 Department of Environmental and Occupational Health Sciences, University of Washington, Seattle, USA; 9 Department of Medicine, University of Washington, Seattle, USA

**Keywords:** Hand hygiene compliance, Plan-do-study-act (PDSA), Low-resource settings, Infection prevention and control, Quality improvement

## Abstract

**Background:**

Hand hygiene (HH) is central in prevention of health care-associated infections. In low resource settings, models to improve HH compliance are needed. We implemented a continuous quality improvement (CQI) program targeting HH in two hospitals in Kenya.

**Objective:**

To determine the impact of the HH CQI program and identify factors associated with HH compliance between 2018 and 2019.

**Methods:**

A CQI project targeting the improvement of hand hygiene was implemented, including training and mentorship. Data were collected monthly between April 2018 and December 2019 in Thika and Kitale Hospitals. Healthcare workers trained on Infection Prevention and Control (IPC) observed and recorded HH opportunities and subsequent compliance among staff, including nurses, clinicians, and auxiliary staff, using the World Health Organization’s “*My Five Moments for Hand Hygiene*” tool. Covariates were explored using mixed-effects logistic regression with random department-level intercepts.

**Results:**

Hand hygiene compliance improved from 27% at baseline to 44% after 21 months. Indication/moment for HH was significantly associated with compliance. Adjusting for site, professional category and department, compliance was higher after a moment of body fluid exposure (aOR 1.43, 95% CI 1.17–1.74, *p* value < 0.001) and lower before an aseptic procedure (aOR 0.12, 95% CI 0.08–0.17, *p* value < 0.001) compared to after patient contact. Wearing of gloves often replaced proper HH in surgical departments, which although not significant, had lower compliance compared to departments for internal medicine (aOR 0.93, 95% CI 0.85–1.02). Adjusted HH compliance from all quarters improved from baseline, but comparing each quarter to the previous quarter, the improvement fluctuated over time.

**Conclusion:**

Training and mentorship on the importance of HH for all moments is needed to improve overall HH compliance. CQI with regular monitoring and feedback of HH performance can be an effective approach in improving HH compliance in public hospitals in Kenya.

## Introduction

Healthcare associated infection (HAI) is a major problem for patient safety. An estimated 5–15 of every 100 hospitalized patients in developed countries acquire HAI while in hospital [1]. This is associated with prolonged hospital stay and additional treatment, increased healthcare costs, long-term disability and excess mortality [1, 2]. The burden of HAI is estimated to be even higher in developing countries, with prevalence rates varying between 14.8 and 19.1% [1]. The prevalence of HAI in low and middle income countries is unknown, as HAI surveillance is limited in these settings [3]. The most common source of HAI transmission is through contaminated healthcare worker (HCW) hands [4–7]. This can be attributed to limited infrastructure to support infection prevention control measures including a constant supply of running water for handwashing or ABHR [8, 9].

Effective hand hygiene is a critical element in reducing the incidence and transmission of HAI [10, 11]. Improving Hand hygiene (HH) compliance alone has been reported to reduce HAI incidence by up to 31% [12]. However, poor HH compliance has been reported in many clinical settings [13]. In Sub-Saharan Africa, a systematic review reported a pooled HH compliance rate of 21%, which is lower than the 40% HH compliance rate observed in developed settings [13]. Factors associated with low HH compliance include HCWs understaffing with competing patient priorities, which limits the time they can spare for HH [2, 11, 14], low belief in the effectiveness of HH in reducing infection transmission [15], poor HH role modelling by other HCWs, inadequate supplies for HH, in particular, alcohol based hand rub (ABHR) and liquid soap [16] and lack of organizational support [11]. HH compliance varies by HCW professional category, department and indication (i.e., before patient contact, before performing an aseptic/clean procedure, after body fluid exposure risk, after touching a patient, and after contact with patient surroundings). HH compliance is higher after patient contact compared to before patient contact which could be attributed to self-protection [17]; doctors are associated with poorer HH compliance compared to other professional healthcare worker categories [18]; and some departments, including intensive care units (ICU) have low HH compliance rates, presumably due to the multiple and repeated HCW-patient contact episodes, resulting in increased workload per staff [19].

A continuous quality improvement (CQI) approach can be an effective strategy towards improving HH compliance. CQI is a progressive process of measurable improvement in the efficiency, effectiveness of performance outcomes in services or processes, to improve patient care [20]. The CQI approach has been used to improve HH practices elsewhere [21, 22]. Using a plan-do-study-act (PDSA) cycle, low-cost high-impact interventions with continuous HH compliance monitoring can be applied iteratively in a clinical setting to improve HH compliance. However, little is known about the impact of this approach in public hospitals in sub-Saharan Africa. This analysis describes the results of a HH CQI program implemented in two hospitals in Kenya between 2018 and 2019, identifying factors associated with HH compliance.

## Methods

### Study design and setting

The project was implemented in two public regional hospitals: Kitale county referral hospital in Trans Nzoia county and Thika level 5 hospital in Kiambu county, with a 250 and 300 bed-capacity, respectively. They each have a hemodialysis unit and Thika has an ICU. The emergency rooms (ER) and outpatient departments in Thika and Kitale hospital see a daily average of 960 and 750 patients, respectively. In addition, both hospitals also have ambulatory specialist clinics, a maternal child health (MCH) clinic and laboratory. The HH interventions were part of a comprehensive implementation of infection prevention and control (IPC) measures. The study design for the quality improvement data collection consisted of repeated cross-sectional observations over time.

### Intervention

**Fig. 1:**
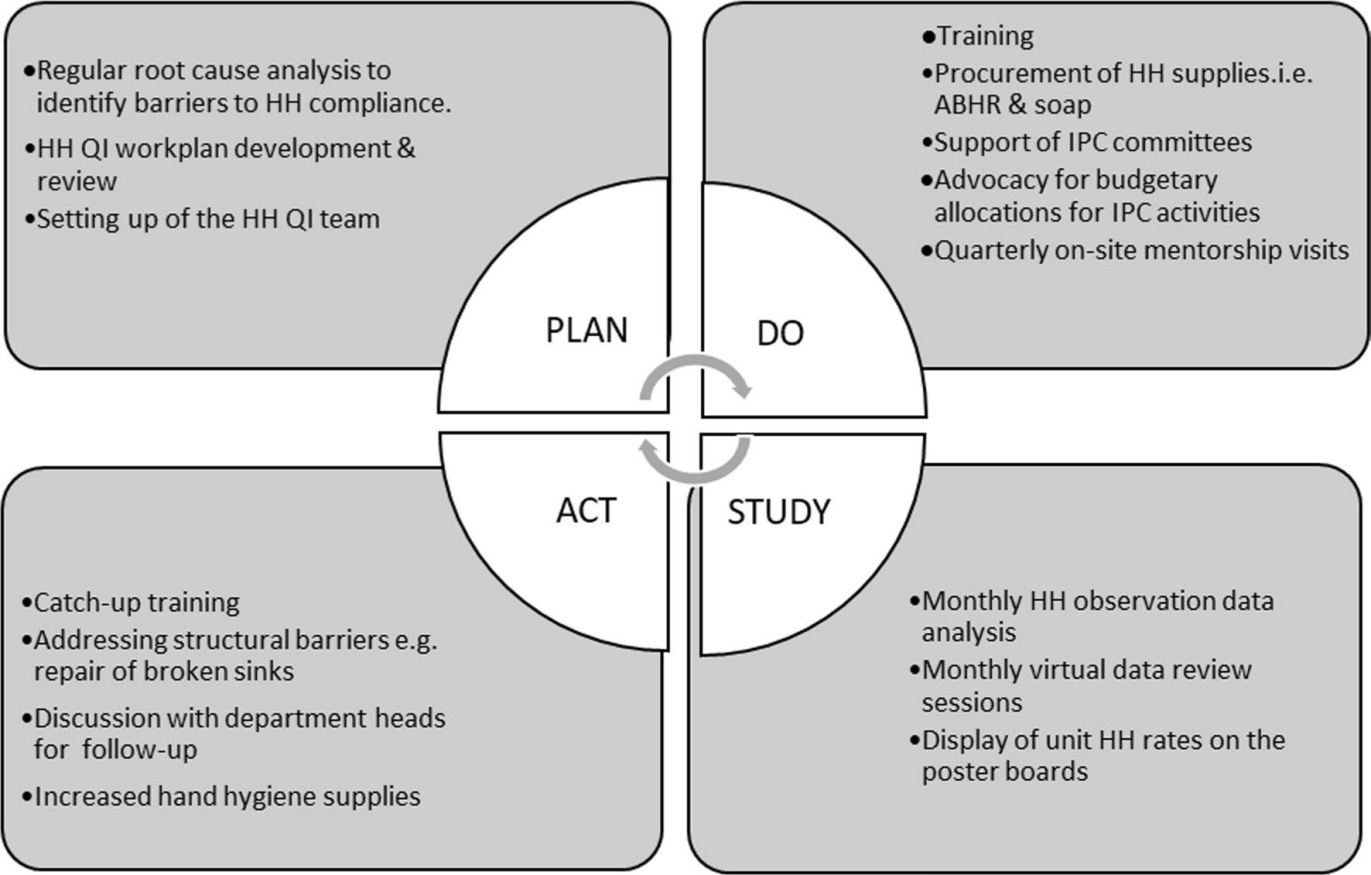
PDSA CQI framework for HH improvement

In 2017, the University of Washington’s International Training and Education Center for Health (I-TECH) Kenya, together with the Ministry of Health (MoH) in collaboration with the County Health Departments, with support from the US Centers for Disease Control and Prevention (CDC), introduced a HH compliance improvement program, based on the PDSA cycle, as part of IPC activities at these two hospitals (Fig. 1).

In the planning phase, the hospitals identified HH as an IPC area they could target for improvement using a CQI approach. Using a root-cause analysis and driver diagram exercises [23], the main barriers to HH compliance were identified, including poor knowledge on all the indications for HH as well as its importance; limited infrastructure as most of the wards and service areas only had one handwashing sink located at the nurses’ station, and it did not always have running water; lack of supplies such as liquid soap and ABHR; and absence of a formal IPC program within the hospitals. I-TECH supported the appointment of IPC focal persons and the setup and running of the IPC committees at each hospital. I-TECH also conducted training on CQI methodology with IPC committee members and other HCWs, developing work plans for HH compliance improvement to foster a sense of ownership of the program.

In the “do” phase, I-TECH and MOH addressed these barriers by providing an initial formal training on general IPC using an MOH-approved 6-day curriculum to a critical mass of HCWs in the two hospitals. All the cadres of HCWs were involved, except students, who were given a one-day orientation in IPC at the start of their clinical rotations. Initial ABHR stock was procured for the hospitals to get them started on the QI project. Finally, the IPC committees were strengthened and supported to conduct regular review meetings as well as advocate for adequate and timely budgetary allocations for IPC activities from the hospital management team, including following up on repair of faulty taps and sinks. The facilities were also supplied with posters at each sink on the 5 moments of HH.

In the “study” phase, IPC committee (IPCC) members were then trained on HH compliance audit using the WHO HH checklist, monitoring the five moments/opportunities for HH [24]. Using the WHO HH checklist [25], IPCC members observed fellow HCWs within the departments as they performed their regular duties, indicating if HH was performed when there was an opportunity (Fig. 1). They tried to be as unobtrusive as possible as they made the observations. compliance was by either handwashing with soap and running water or use of ABHR. Gloving in place of handwashing/ABHR use was recorded and considered non-compliant.

HH compliance audits were done on different days/times of the week but limited to day-time hours between Monday and Friday, with each hospital making at least 400 observations per month. IPCC members observed staff with direct patient contact, including primary physicians and consultants, nurses and midwives, physiotherapists, biomedical technicians and engineers, laboratory personnel, nutritionists, students from various medical professional categories and auxiliary staff. HH compliance audits were conducted from April 2018 and were on-going as of May 2021.

These data were collected on paper forms and transcribed into a macro-enabled spreadsheet which ran simple trends and calculated HH compliance proportions by department, professional category and indication. These were then summarized monthly by I-TECH technical advisors, with feedback to the IPCC members through monthly virtual meetings, which MOH and CDC technical officers also attended. Gradually, IPCC members took up the analysis and interpretation of the monthly data, leading the monthly data review meetings with cross-sharing across the two hospitals. A summary report was shared with the hospital administration. Departments also displayed their monthly compliance rates on their notice boards.

In the “action” phase, following the data audit and feedback, HCWs identified the barriers to HH compliance and developed micro-level action plans on how to address them (e.g., if in a certain department reduced HH compliance was attributable to a broken sink they followed it up with maintenance to ensure it got fixed). Other actions included talking to department heads and sharing the HH compliance graphs for further intra-departmental follow-up. These data were also shared with the hospital management team and sub-national government departments of health and were used as evidence to advocate for budgetary allocation for IPC supplies.

In the four subsequent PDSA cycles, other intervention actions included on the job training sessions at the unit level (including auxiliary workers); monthly mentorship sessions where I-TECH officers provided topic-based technical assistance; biweekly data review and feedback sessions through video calls; and quarterly site visits by I-TECH and MOH for further mentorship.

### Data analysis

HH compliance audit data collected between April 2018 and December 2019 were used, in a panel data format, with repeat observations from the same departments over time.

Descriptive statistics and HH compliance were summarized using frequencies and proportions, stratified by HH indication, professional category, department and hospital. Departments were categorized as medical, surgical, obstetrics, and gynecology (including labor and delivery, newborn unit), ambulatory (specialist clinics, MCH clinic, laboratory), outpatient (including ER) and critical care (ICU, hemodialysis units).

Exploratory univariable analysis by professional category, department and time (parameterized as annual quarters) was performed. Multilevel models using mixed effects logistic regression with random department-level intercepts were used to estimate odds ratios (ORs) for hypothesized correlates of HH compliance. Given that individuals observed were not uniquely identified, we nested observations at the department level to address correlation since HCWs could have been observed multiple times each month as they remained within the department. ORs and 95% confidence intervals (CIs) were reported. Analyses were conducted using STATA 15. (StataCorp. 2017. Stata Statistical Software: Release 15. College Station, TX: StataCorp LLC.)

## Results

A total of 12,923 HH observations over 21 months (7 quarters) were included in the analysis. The indication with the highest number of observations was after patient contact with 4455 observations (34.5%) while the lowest was after body fluid exposure with 810 observations 6.3%), as shown in Table 1. Most of the observations were from Thika (7429 [57.5%]) and the largest proportion of HCWs observed were nurses (4227 [32.7%]).

**Table 1:** Site characteristics of the two hospitals implementing a HH project in Kenya, 2018–2019

	Kitale n (%) (N = 5494)	Thika n (%) (N = 7429)	Total n (%) (N = 12,923)
1. Indication			
After patient contact	1312 (23.9)	3143 (42.3)	4455 (34.5)
Before patient contact	1432 (26.1)	2218 (29.9)	3650 (28.2)
After touching the patient surrounding	1339 (24.4)	992 (13.4)	2331 (18.0)
Before aseptic procedure	794 (14.5)	883 (11.9)	1677 (13.0)
After body fluid exposure	617 (11.2)	193 (2.6)	810 (6.3)
2. Department			
Internal medicine^a^	1806 (32.9)	1619 (21.8)	3425 (26.5)
Surgery^b^	1439 (26.2)	1095 (14.7)	2534 (19.6)
Obstetrics and gynecology^c^	1219 (22.2)	1571 (21.1)	2790 (21.6)
Ambulatory^d^	345 (6.3)	1220 (16.4)	1565 (12.1)
ER, OPD	407 (7.4)	1010 (13.6)	1417 (11.0)
ICU, hemodialysis unit	278 (5.1)	914 (12.3)	1192 (9.2)
3. Professional category			
Nurse/midwife	1976 (36.0)	2251 (30.3)	4227 (32.7)
Medical doctor^e^	1807 (32.9)	1688 (22.7)	3495 (27.0)
Student^f^	1300 (23.7)	2259 (30.4)	3559 (27.5)
Other health care worker^g^	163 (3.0)	507 (6.8)	670 (5.2)
Auxiliary^h^	248 (4.5)	724 (9.7)	972 (7.5)
4. Time in quarters (Qr)			
Qr 0 (April–June 2018)	348 (6.3)	507 (6.8)	855 (6.6)
Qr 1(July–September 2018)	461 (8.4)	1166 (15.7)	1627 (12.6)
Qr 2 (October–December 2018)	1234 (22.5)	1267 (17.1)	2501 (19.4)
Qr 3 (January–March 2019)	226 (4.1)	771 (10.4)	997 (7.7)
Qr 4 (April–June 2019)	1062 (19.3)	1189 (16.0)	2251 (17.4)
Qr 5 (July–September 2019)	1125 (20.5)	1279 (17.2)	2404 (18.6)
Qr 6 (October–December 2019)	1038 (18.9)	1250 (16.8)	2288 (17.7)

ER, emergency room; OPD, outpatient department; ICU, intensive care unit

Departments: ^a^female medical ward, male medical ward and pediatrics ward; ^b^female surgical ward, male surgical ward and operating theatre; ^c^gynecology ward, maternity, labor and delivery unit, newborn unit; ^d^dental clinic, diabetes clinic, maternal, child health and family planning department (MCH& FP), physiotherapy clinic, laboratory

Professional subcategories: ^e^house officers, physicians, surgeons, dentists, pediatricians, gynecologists, clinical officers; f– medical students, nursing students, dental students and other students; ^g^dieticians, physiotherapists; ^h^housekeeping staff, technicians, engineers

Overall baseline HH compliance was 27% which cumulatively improved to 44% after nearly two years (Table 2). The moments after body fluid exposure had the greatest percentage change (43%), while those before an aseptic procedure had the lowest compliance both at baseline (14%) and post intervention (19%).

**Table 2:** Hand hygiene compliance comparing the first quarter to subsequent quarters two hospitals implementing a HH project in Kenya, 2018–2019

Characteristic	Baseline (Qr 0) n = 855		Post intervention (Qr 1–6) n = 12068		% Change
Overall compliance	229/855	27%	5329/12068	44%	17%
Indication					
After patient contact	83/230	36%	2748/4225	65%	29%
Before patient contact	50/268	19%	854/3382	25%	7%
After touching the patient surrounding	56/164	34%	897/2167	41%	7%
Before aseptic procedure	16/114	14%	296/1563	19%	5%
After body fluid exposure	24/79	30%	534/731	73%	43%
Department					
Internal medicine	43/233	19%	1258/3192	39%	21%
Surgery	18/158	11%	886/2376	37%	26%
Obstetrics and gynaecology	68/228	30%	1123/2562	44%	14%
Ambulatory	31/79	39%	723/1486	49%	10%
ER, OPD	34/87	39%	592/1330	45%	5%
ICU, hemodialysis unit	35/70	50%	747/1122	67%	17%
Professional category					
Nurse/midwife	104/390	27%	2004/3837	52%	26%
Medical doctor	56/245	23%	1242/3250	38%	15%
Student	39/102	38%	1414/3457	41%	3%
Other healthcare worker	19/79	24%	285/591	48%	24%
Auxiliary	11/39	28%	384/933	41%	13%
Site					
Kitale	57/348	16%	2038/5146	40%	23%
Thika	172/507	34%	3291/6922	48%	14%

(%)—row percentages

ER, emergency room; OPD, outpatient department, ICU, intensive care unit

Of note, 71% of the moments before an aseptic procedure had the highest number of observations marked by gloving without any handwashing or use of ABHR. Across the departments, the ICU and Hemodialysis Unit had the highest compliance post intervention at 67%, but the surgical department had the greatest percentage change at 26% (Table 2). Compliance ranged between 38 and 52% across the professional categories, with nurses/midwives having the greatest percentage change (26%). While Thika had a higher compliance rate at 48%, Kitale’s percentage change was nearly twice as much as that observed in Thika (23% vs 14%).

When assessing factors associated with HH compliance, the nature of the HH opportunity was significantly associated with HH compliance. Adjusting for professional category, hospital, department and time (quarter of assessment), HH compliance was 43% higher after a moment of body fluid exposure compared to after patient contact (aOR 1.43, 95% CI 1.17–1.74, *p* value < 0.001). HH was 0.12 times less likely to be observed before an aseptic procedure (aOR 0.12, 95% CI 0.08–0.17, *p* value < 0.001) (Table 3).

**Table 3:** Factors associated with HH compliance in two hospitals implementing a HH project in Kenya, 2018–2019

Characteristic	Unadjusted OR	(95% CI)	*p* value	Adjusted OR	(95% CI)	*p* value
Indication						
After patient contact	Ref	–	–	Ref	–	**–**
Before patient contact	0.18	0.15–0.22	< 0.001	0.17	0.14–0.22	**< 0.001**
After touching the patient surrounding	0.39	0.33–0.46	< 0.001	0.42	0.34–0.50	**< 0.001**
Before aseptic procedure	0.13	0.08–0.20	< 0.001	0.12	0.08–0.17	**< 0.001**
After body fluid exposure	1.33	1.09–1.63	0.005	1.43	1.17–1.74	**< 0.001**
Department*						
Internal medicine	Ref	–		Ref	–	
Surgery	0.91	0.81–1.00		0.93	0.85–1.02	
Obstetrics and gynaecology	1.22	1.10–1.35		1.12	1.03–1.22	
Ambulatory	1.51	1.34–1.71		1.52	1.36–1.69	
ER, OPD	1.29	1.14–1.46		1.26	1.12–1.41	
ICU, hemodialysis unit	3.11	2.71–3.57		3.28	2.88–3.73	
Professional category						
Nurse/midwife	Ref	–	–	Ref	–	**–**
Medical doctor	0.66	0.49–0.87	0.003	0.58	0.41–0.83	**0.003**
Student	0.76	0.64–0.90	0.002	0.67	0.54–0.83	**< 0.001**
Other healthcare worker	0.82	0.43–1.53	0.52	0.91	0.33–1.50	0.365
Auxiliary	0.60	0.48–0.75	< 0.001	0.5	0.40–0.63	**< 0.001**
Hospital						
Kitale	Ref	–	–	Ref	–	**–**
Thika	1.25	0.99–1.58	0.063	1.28	0.96–1.70	0.093
Time in quarters						
Qr 0 (baseline)	Ref	–	–	Ref	–	**–**
Qr 1	1.93	1.46–2.55	< 0.001	2.02	1.43–2.86	**< 0.001**
Qr 2	2.77	1.76–4.36	< 0.001	3.34	1.92–5.80	**< 0.001**
Qr 3	2.65	1.67–4.19	< 0.001	3.01	1.65–5.50	**< 0.001**
Qr 4	1.53	1.21–1.94	< 0.001	1.79	1.28–2.49	**0.001**
Qr 5	2.10	1.35–3.26	0.001	2.41	1.41–4.12	**0.001**
Qr 6	2.38	1.52–3.73	< 0.001	2.70	1.59–4.61	**< 0.001**

Bold *p*-values are statistically significant

ER, emergency room; OPD, outpatient department; ICU, intensive care unit

*Adjusted for department as a random effect variable with confidence intervals calculated from standard error of variance, hence *p* values are unavailable

Professional category was also significantly associated with HH compliance. Compared to nurses, students were 33% (aOR 0.67, 95% CI 0.54–0.83, *p* value < 0.001) and doctors were 42% (aOR 0.58, 95% CI 0.41–0.83, *p* value 0.003) less likely to be compliant. Auxiliary workers were half as likely to be compliant compared to nurses (aOR 0.50, 95% CI 0.40–0.63, *p* value < 0.001).

All the departments had higher compliance compared to Internal Medicine, except for Surgery. HH compliance was 7% lower in Surgery compared to Internal Medicine, though this was not statistically significant (aOR 0.93, 95% CI 0.85–1.02), and 3 times as high in ICU/Hemodialysis Unit (aOR 3.28, 95% CI 2.88–3.73) compared to Internal Medicine.

While Thika had 28% higher compliance compared to Kitale, this was not statistically significant. (aOR 1.28, 95% CI 0.96–1.70, *p* value 0.093).

**Fig. 2:**
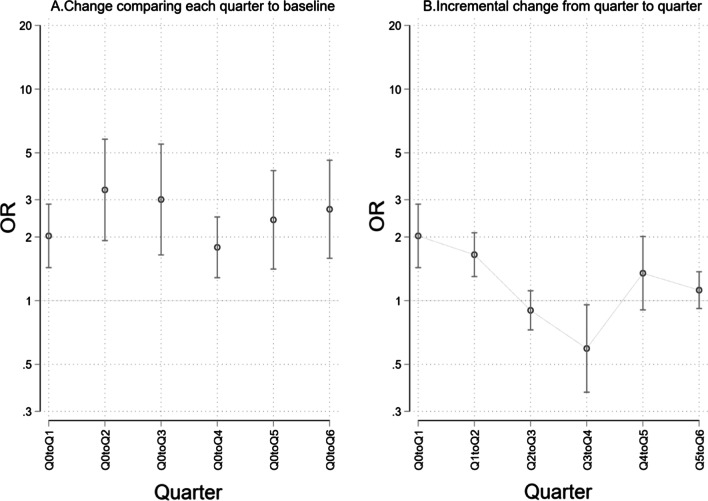
Change in HH compliance comparing **A** each quarter to the baseline quarter and **B** from quarter to quarter. Q0toQ1—baseline to quarter 1; Q1toQ2—quarter 1 to quarter 2; OR—odds ratio

HH compliance improved with time. Adjusting for indication, site, professional category and department, all subsequent quarters were more likely to have higher compliance compared to the first quarter (Fig. 2).

Assessing change from quarter to quarter, the improvement was not constant, as shown in Fig. 2B.

**Fig. 3:**
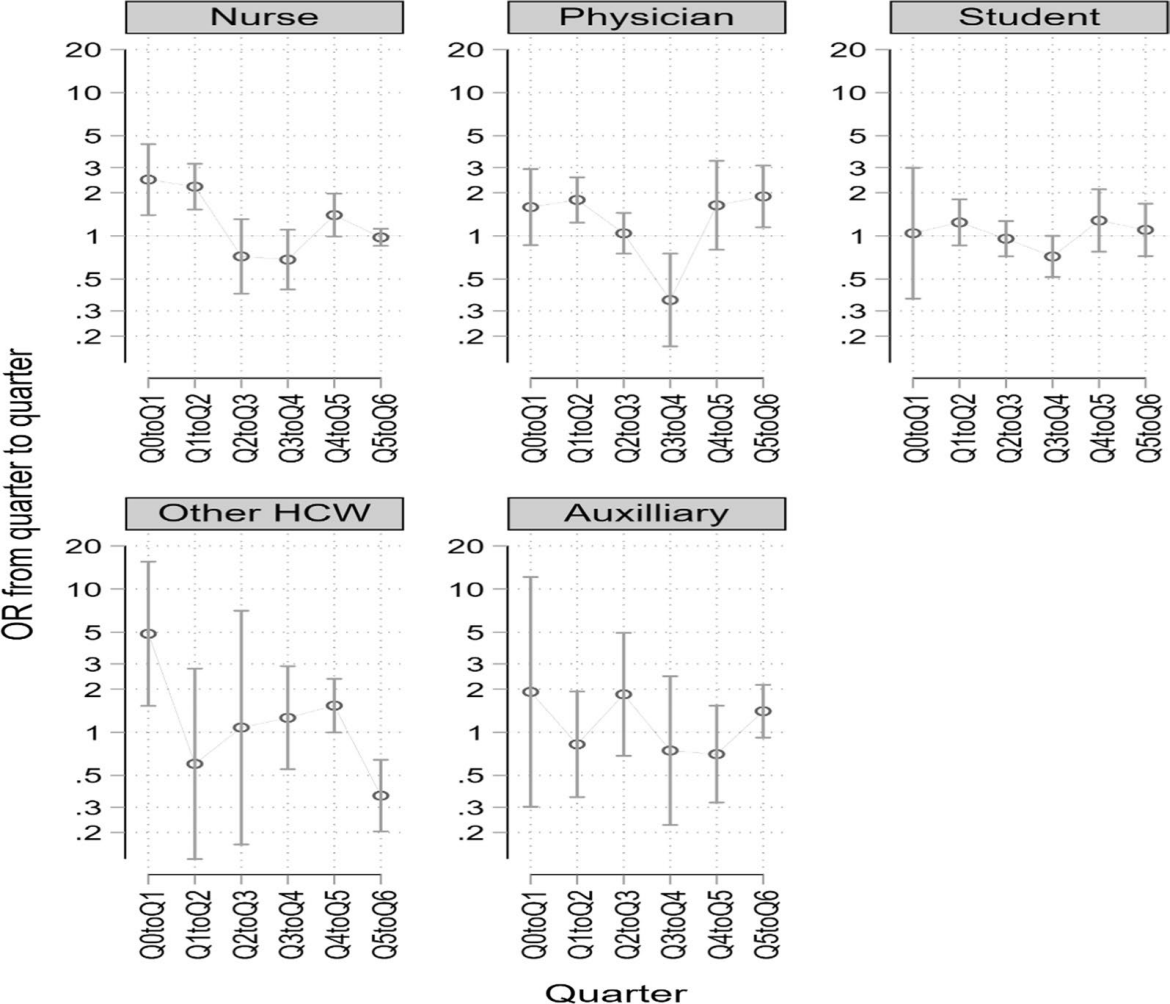
Change in adjusted OR over time stratified by professional category. Other HCW—dieticians, physiotherapists; Auxiliary staff—housekeeping staff, technicians engineers; Q0toQ1—baseline to quarter 1; Q1toQ2—quarter 1 to quarter 2; OR—odds ratio

Stratifying this improvement over time from quarter to quarter across professional categories, the trend was fairly similar between doctors and nurses (Fig. 3). Students do not show significant improvement, as their trend is clustered around the null line, while other healthcare workers and auxiliary staff show the most variation (Fig. 3).

## Discussion

Following implementation of a multimodal HH improvement program using a quality improvement approach, we observed a modest 17% improvement in HH compliance comparing the baseline quarter (27%) to subsequent 6 quarters (44%). This is consistent with other studies in similar low income settings: 13% improvement in HH compliance in Ethiopia [26], 10% in Kenya [27] and 23% in Iran [28], even though the intervention in each study involved different approaches of improving HH.

Consistent with other studies, compliance was highest when the indication for HH was after HCW exposure to potential contaminants [after body fluid exposure (73%), after patient contact (65%) and after touching the patients’ surroundings (41%)] relative to indications prior to patient contact [29–31]. This can be explained by the preponderance of “inherent” HH behavior, where HH is performed because a person perceives their hands to be dirty and so observes HH for self-preservation, even outside of the healthcare setting [32]. More so, we observed high prevalence of gloving among indications prior to patient contact, implying that HCW tend to falsely interpret use of gloves as a form of HH. Further HCW mentorship and emphasis on HH compliance for patient safety is therefore needed, to improve compliance among indications prior to patient contact.

We observed the highest compliance in ICU and Hemodialysis departments compared to the Internal Medicine department. Findings from other studies on HH compliance in ICU are mixed. Some studies have reported lower compliance in ICU relative to other departments [33–35] due to a high number of HH opportunities per patient given their moribund state. This coupled with understaffing leads to patients’ needs taking priority over HH. However, other studies have reported higher compliance attributable to the staff’s heightened self-awareness on the need to observe HH due to the critical nature of ICU patients [36]. Similarly, the accident and emergency department and outpatient departments, which have high patient volumes at a time, with frequent contact between HCWs and patients, had relatively higher compliance compared to the internal medicine department, not dissimilar to findings by others [37]. This could be explained by the fact that in the A&E department, there is likely more infrastructure per square area supporting HH, e.g. hand washing sinks and mounted hand sanitizers compared to the surgical and gynecology departments where the sinks are limited to one corner within the ward and the hand sanitizers are mostly available during procedure hours on the work station trolleys and at the nurses’ station. More so, there is likelihood of frequent gloving within the surgical and gynecological departments, with the incorrect assumption that this obviates the need for HH.

Compliance in each professional category, except for nurses/midwives, was below 50%. Medical doctors had 42% lower compliance compared to nurses, a common reported observation [13, 17]. We hypothesize that this could be due to the brief doctor-patient contact time relative to nurse-patient contact time, hence doctors do not perceive themselves as posing a risk of HAI transmission to patients [38]. Another reason could be the higher coverage of IPC training and interventional programs among nurses relative to all other cadres, as they form the largest proportion of healthcare professionals in these hospitals. However, these findings on compliance by professional category ought to be interpreted with caution, as it could be a statistical artifact given that observations among doctors are relatively fewer compared to those among nurses, with one doctor likely contributing more observations per session compared to one nurse. This is supported by the near-similar trends seen in Fig. 3, when trajectories are compared over time within each professional category. The lack of improvement in compliance rates among students over time calls for a full IPC induction program among students upon deployment to the clinical areas and consideration of students’ involvement in hospitals’ IPC training sessions where they have the opportunity to learn alongside their clinical preceptors, an initiative that could contribute to making them more accountable towards the hospital IPC improvement program.

Improvement in compliance over time may have been a result of the cyclical nature of the program. Data were analyzed, reviewed and disseminated monthly in conjunction with the sites, where they would identify the gaps as highlighted by the data. The facility IPC committee would then develop action plans on addressing the gaps and maintaining the factors promoting good performance, which would be reviewed routinely. Of note, the number of observations in 2019 was almost twice that in 2018, which could imply an increase in the observation acumen of the HH observers. This is evidence of the effectiveness of on-job training and mentorship they received over time. The decline in HH compliance performance noted between the second and third quarters (Fig. 2B) could be explained by an industrial action by nurses at the time, whose effect then lagged on to the fourth quarter. This finding further underpins the need for continuous support in the implementation of CQI projects over time, as the improvement is not always linear, due to effects of external factors outside of the project. Such improvements can easily be missed if periods of support and observation are short.

Our findings are consistent with similar studies using the CQI approach in low resource settings. Implementation of a similar program in Ghana led to improvement in HH compliance from 67 to 92% [21] and was useful in guiding system-wide interventions for patient safety in Brazil [39].

One of our study strengths was the large number of observations collected over a long period of time, improving the precision of our findings. This was also done in a routine health service delivery setting as opposed to a controlled research setting, making our findings generalizable to other similar settings. However, we were limited in that we did not have a control group that did not receive the CQI program. Despite observers being as inconspicuous as possible, residual “Hawthorne effect” could be biasing the improvement estimates. Another limitation is that while the unit of analysis is observations, several observations could have been contributed by one individual; therefore, when adjusting for professional category, weighting based on the number of individuals observed would have been ideal. Lastly, data on functional hand hygiene stations at the time of the compliance assessments is not available and would have been complementary to these findings.

## Conclusion

Implementation of a continuous quality improvement approach with regular monitoring of HH performance and feedback could modestly improve HH compliance in public hospitals within resource-limited settings. Given the level of effort in setting up and maintaining such CQI projects, long-term studies are warranted to assess whether such IPC practices are sustained beyond the project. Low rates of compliance before patient contact highlight the role of HCWs hands in transmitting and conversely preventing hospital acquired infections and call for emphasis on this in training and mentorship programs.

## Data Availability

The datasets used and/or analyzed during the current study are available from the corresponding author on reasonable request.

## Abbreviations

CQI: Continuous quality improvement
PDSA: Plan-do-study-act
HH: Hand hygiene
HAI: Health care-associated infections
HCW: Healthcare worker
ICU: Intensive care units
ER: Emergency rooms
MCH: Maternal child health
IPC: Infection Prevention and Control
I-TECH: International Training and Education Center for Health
MoH: Ministry of Health
CDC: Centers for Disease Control and Prevention
ABHR: Alcohol-based hand rub
IPCC: Infection Prevention and Control Committees
WHO: World Health Organisation
OR: Odds ratios
CI: Confidence intervals
STATA 15: StataCorp. 2017. Stata Statistical Software: Release 15. College Station, TX: StataCorp LLC
